# Psora and Syphilis

**Published:** 1885-03

**Authors:** 


### PSORA AND SYPHILIS.

DR. WOLF.

The greatest evil of psora is that it reproduces itself and descends to posterity. The children of psoric parents are very often born with *malformations*, among which tumors of the head and yellowish, sallow color of the skin, old face, head too large, phthisical conformation, hernia, clubfoot, etc., may be mentioned. The children are often born with *scabies*, which will infect the whole family: they soon get scald-head and other eruptions. It is, therefore, the duty of every family physician to begin the antipsoric treatment as early as possible in newly-married people, for during pregnancy the cure progresses but very unsatisfactorily. Psora has, in the course of time and under favorable circumstances, reached its culminating point, and produced a new form of disease toward the end of the fifteenth century—viz.: *syphilis*.

The connection of psora and syphilis is proved by the fact that since the appearance of syphilis the lepra has become very scarce. The syphilitic dyscrasia has three peculiar characteristics: First, want of flexibility of the limbs, which refuse to obey the impulse of the will—a painful crepitating noise in the joints when moved accompanies it; second, shuddering when going to stool, and, third, sleeplessness without apparent cause.

Primary syphilis will, under favorable conditions, remain stationary for twenty years, which has been observed by me in several cases. But if the chancre is interfered with, and if Mercury in large doses is used and the ulcer healed by force, the *slow chancrous dyscrasia* is the general result.

The outbreak of secondary symptoms is in most cases caused by taking a severe cold, often showing itself in an attack similar to acute gout. The combination of the syphilitic and the mercurial poisons greatly aggravates all pre-existing morbid dispositions; the liability to take cold is greatly augmented; the worst form of coryza, with stinking, corroding, secretion, is produced; the glands are affected, and a great disposition to parenchymatous inflammations, ulcerations, dissolution of the blood, etc., etc., is produced.

But all this suffering Mercury alone can produce without its combination with syphilitic poison. Mercury and Iodine are the *very worst poisons*, and should never be used but in syphilis. The affection of the mucous membrane, so often treated by Mercury, will also yield to *Apis*. Scrofula, tubercles, tumor, and goitre will yield to *Thuja*. Only against syphilis *Mercury* and *Iodine* are indispensable.

Pure syphilis requires Mercurius; the combination of syphilis and sycosis requires *Iodine*, only one single dose in the thirtieth potency. When a cure is not effected by it, it is a sure sign that one of the following three impediments hinders the cure:

1. Abuse of Mercury or Iodine; the first can be removed by one dose of Mercury 6,000, the second by one dose of Iodine 5,000 potency. Where both have been abused, it is necessary to give, first, Mercury 6,000, and afterward Thuja 1,000.

2. The second impediment is the predominating influence of the *psoric* poison. This requires one dose of *Sulphur*$^{30}$, or in case of abuse of Sulphur the 6,000th potency must be used (if the previous dose of Mercurius$^{30}$ has left the cure unfinished).

3. The third impediment is the predominating influence of the *sycotic* poison, the treatment of which will be given under Sycosis. Syphilitic ulcers require Kali-bich.$^{30}$, one dose, where the cure remains unfinished; after it, Sanguinaria $^{30}$.

Sanguinaria$^{200}$ is *the* remedy for that severe one-sided headache extending into the sinus-frontalis which *Quinine* never cures. Corrosive sublimate will remove it quickly, but it generally returns after some time in an aggravated form.

Where the blood has already a great tendency to dissolution, with great want of strength, suggillation of the blood, bleeding from the nose, lungs, or intestines, with a scorbutic state of the gums, *Nitric acid* is the proper remedy in chronic cases, one dose of the 30. In more acute cases, one dose 30 every twenty-four hours for three days, and in the worst cases the 30 in dilution every one to three hours till amelioration sets in.

Inflammation of the lungs on syphilitic ground is also to be treated by *Nitric acid,* and where this does not suffice, Sanguinaria$^{200}$, every three hours. Nitric acid is also the principal remedy in that bad form of disease of the throat with swelling of the mucous membrane ending at last in "phthisis laryngea." Where this does not suffice, one dose of *Apis*$^{30}$, and afterward *Fluor-acid.* Fluor-acid$^{30}$ in the milder cases; in the most serious cases, the 2,000, one dose in *five days.*

Where the syphilitic poison has concentrated itself on the liver and consensually affects the spleen, kidneys, and genital organs, Lycopod.$^{200}$ is the proper remedy. Mag. mur. and Natrum-mur. only aggravate the symptoms in such cases, even where they seem to correspond with the symptoms, and this explains why the sea-bath is so injurious in syphilitic diseases. Lycopod. is also the best remedy in those dangerous hemorrhages of the womb in syphilitic and mercurial cachexia, the 200 potency every three hours, and the same remedy holds good in those cases of bloody urine and in hypochondriasis and hysteria originating in the above stated combination of the syphilitic and mercurial poisons.

Syphilitic affections of the nose require *Aurum*$^{200}$, a dose every twenty-four hours for seven days. Exostosis and tophus are only produced by Mercury in large doses, therefore Mercury$^{6000}$ must be given. Where caries has already set in, and the cure does not progress after that dose of Mercury, Silicea$^{30}$ is to be given, as neither higher nor lower potencies will succeed. Where, in the worst cases, Silicea cannot accomplish the cure, one dose of Sulphur$^{30}$ is yet required. Where the bone-pains do not quickly yield to Mercury, Apis$^{3}$, in water, must be given.

Softening of the bones, swelling, and curvature yield to Fluor-acid$^{2000}$, one single dose. Brittleness, desiccation, and breaking of the bones yield to Calc. carb.$^{200}$, in water, one dose daily for five days.

The third impediment to the cure of syphilis is the predominating influence of sycosis. The sycotic poison is the result of a combination of psora and syphilis in their highest potency. It is a dyscrasia which has spread fearfully, and in a hitherto inexplicable manner, since the beginning of the present century, so much so, that if this progression should continue on at the same rate, the very existence of mankind is in jeopardy.

The sycotic poison greatly increases the disposition to all those every-day illnesses, and it renders all diseases more obstinate and pernicious.

Dr. Wolf then gives a minute description of the progress of the disease and of the manner in which by degrees the different organs are affected by the poison. Only a short list of the principal lesions can be given here.

Affections of the teeth, with loosening of the roots and falling out, with the most obstinate form of prosopalgia, alternating with the most insufferable cephalalgia. Affections of the mouth with cracked lower lip, peeling of the epithelium and small white, flat ulcers, etc.

Hypochondriasis; pains in the muscles; spasms; giddiness; deadness of the tips of the fingers and toes; constipation; breath smelling like carrion; affections of the mucous membrane and infectious character of its secretions; tubercles; warts; fungous excrescences; varicose veins; deposition of bacon-like fat; gout; chronic catarrh of the urinary organs; Bright's disease; diabetes, etc.

The small-pox is the efflorescence of the sycotic poison, and hence vaccination is the greatest aberration of the human mind. The most prominent symptoms of the sycotic dyscrasia, after clap and leucorrhœa, without any previous affection, are sometimes observed as the result of vaccination. Likewise we often find great disposition to self-pollution, affections of the testicles, ovaries, eyes, ears, teeth, and hair; weakness of the nerves and brain, giddiness, paralytic affections, spasms, asthma, chlorosis, anomalies of menstruation, diabetes, tuberculosis, etc., all appear as an immediate consequence of vaccination, and most of the above-named diseases are the standing and predominating diseases of the present age—influenza, typhus, and whooping cough, with great tendency to tuberculosis have also become standing diseases in a hitherto unheard-of manner.

Progressive paralysis is also a consequence of the continual poisoning of successive generations by the vaccine or sycotic poison. The so-called Egyptian ophthalmia has become a standing disease among the soldiery, and very often follows vaccination; and it is well known that gonorrhœal ophthalmia and the above-mentioned form bear the closest resemblance.

Dr. Wolf then gives a minute proving of Thuja instituted on himself and more than one hundred persons of every sex and age, which contains one thousand and fifty symptoms. The principal results of the proving are the following:

1. Irritation of the mucous membrane of the genital organs, extending itself over all organs. 2. Changing of the naturally mild secretion into an acid, corroding, infectious quality. 3.

Over irritation of all the nerves, with tendency to centripetal paralysis. 4. Disturbance of digestion and sanguification, tendency to destruction, dissolution of the fluids and of the whole organism. It will thus be seen that Thuja corresponds in every respect with the sycotic poison, and thus offers itself as a remedy against the following diseases, which are the consequence of the sycotic poison, viz.:

1. The genuine poisonous fig-wart-gonorrhœa, which is always cured by Thuja$^{30}$, one dose. In new cases, five, seven, and fourteen days are required for a cure; old cases, the longest time and the highest potencies, 300 or 1,000. The action of the drug when once given must be left to act undisturbed.

2. The sycotic poison also shows itself in symptoms of constriction of the urethra, with urging to urinate and irresistible desire to self-pollution. This is shown in young girls, especially after vaccination. Thuja is the remedy, one dose.

3. The extension of the poison upward from the mucous membrane of the genital organs produces long-lasting catarrhs of head and chest. These symptoms are all met by Thuja.

4. Progressive paralysis, with painful aching of the muscles and lightning-like lancinating pains, with trembling and want of control of the will over the muscles. Thuja heals all these.

5. Vertigo, with syncope even to falling, finds its true remedy in Thuja.

6. Sleeplessness, without any apparent cause, resists all remedies except Thuja, this cures.

7. Photophobia, amaurosis, partial paralysis of upper lids, squinting, these affections being the result of small-pox or sycotic gonorrhœa, are also cured by Thuja.

8. Deafness without organic lesion, often alternating with acute hearing, inherited by children. Thuja is the homœopathic remedy.

9. The medulla spinalis and ganglionic systems are affected by sycosis—lancinating pains in face, neck, and along the spine; deadness of single parts, inarticulate speech, and where the nerve rajus and glotto pharyngeus are affected we find a want of sensibility and motion in the stomach and intestines, showing itself by absence of appetite and thirst, or else insatiable voracity, resulting from a want of feeling of repletion; tympanitis, hernia, prolapsus uteri, and also of the vagina and of the rectum; most obstinate constipation, paralysis of the urinary organs, impotence; varices, varicocele, hemorrhoidal tumors, black stools. These evils grow daily more and more prevalent, and the otherwise successful remedies, Sulphur, Pulsatilla, Lycopod., and Fluoric acid grow more and more inefficient, but Thuja cures them all.

